# Neuroprotective effect of phospholipase A_2_ from Malaysian *Naja sumatrana* venom against H_2_O_2_-induced cell damage and apoptosis

**DOI:** 10.3389/fphar.2022.935418

**Published:** 2022-10-14

**Authors:** Nur Atiqah Haizum Abdullah, Nur Qisya Afifah Veronica Sainik, Ezalia Esa, Nur Afrina Muhamad Hendri, Muhamad Rusdi Ahmad Rusmili, Wayne C. Hodgson, Mohd Farooq Shaikh, Iekhsan Othman

**Affiliations:** ^1^ Neuropharmacology Research Laboratory, Jeffrey Cheah School of Medicine and Health Sciences, Monash University Malaysia, Bandar Sunway, Selangor, Malaysia; ^2^ Faculty of Medicine, Centre for Tissue Engineering and Regenerative Medicine, Universiti Kebangsaan Malaysia, Kuala Lumpur, Malaysia; ^3^ Haematology Unit, Cancer Research Centre, Institute for Medical Research, National Institutes of Health Malaysia, Shah Alam, Malaysia; ^4^ Department of Electron Microscopy, Institute for Medical Research, National Institutes of Health Malaysia, Shah Alam, Malaysia; ^5^ Kulliyyah of Pharmacy, International Islamic University Malaysia, Kuantan Campus, Kuantan, Malaysia; ^6^ Department of Pharmacology, Monash Venom Group, Biomedical Discovery Institute, Monash University, Clayton, VIC, Australia

**Keywords:** neuroprotection, snake venom phospholipase A_2_, neurodegenerative disease, apoptosis, mitochondria, inflammation

## Abstract

Oxidative stress is one of the factors involved in the pathogenesis of several neurodegenerative diseases. It has been reported that a secretory phospholipase A_2_ known as A2-EPTX-NSm1a has lower cytotoxicity in neuronal cells compared to its crude *Naja sumatrana* venom. In this study, A2-EPTX-NSm1a was tested for its neuroprotective activity on human neuroblastoma cells (SH-SY5Y) differentiated into cholinergic neurons against oxidative stress induced by hydrogen peroxide (H_2_O_2_). H_2_O_2_ treatment alone increased the caspase-3 and caspase-8 activities, whereas pre-treatment with A2-EPTX-NSm1a reduced the activity of these apoptosis-associated proteins. Moreover, A2-EPTX-NSm1a protects the morphology and ultrastructure of differentiated SH-SY5Y cells in the presence of H_2_O_2_. Oxidative stress increased the number of small mitochondria. Further evaluation showed the size of mitochondria with a length below 0.25 µm in oxidative stress conditions is higher than the control group, suggesting mitochondria fragmentation. Pre-treatment with A2-EPTX-NSm1a attenuated the number of mitochondria in cells with H_2_O_2_ Furthermore, A2-EPTX-NSm1a altered the expression of several neuroprotein biomarkers of GDNF, IL-8, MCP-1, TIMP-1, and TNF-R1 in cells under oxidative stress induced by H_2_O_2_. These findings indicate that anti-apoptosis with mitochondria-related protection, anti-inflammatory effect, and promote expression of important markers for cell survival may underlie the neuroprotective effect of A2-EPTX-NSm1a in cholinergic rich human cells under oxidative stress, a vital role in the neuronal disorder.

## Introduction

Neurodegenerative diseases (NDs) are a wide range of neurological conditions caused by progressive structural and functional degeneration of neurons that lead to neuronal death. Neurodegenerative diseases such as Alzheimer’s disease (AD), Parkinson’s disease (PD), Huntington’s disease (HD) and amyotrophic lateral sclerosis (ALS) are associated with many factors such as age, lifestyle, environmental factors and occupational (Ross and Poirier, 2004; Gunnarsson and Bodin, 2019; Marras et al., 2019; Madore et al., 2020). Neurodegenerative diseases can also result from other secondary to other neurological conditions such as stroke, traumatic brain injury, surgical brain injury, and others (Jadhav et al., 2007; Gupta and Sen, 2016; Veldsman et al., 2020). The degeneration of neurons seen in NDs significantly impact the patient as it leads to a reduction of cognitive function and may also alter emotional and behavioral pattern (Levenson et al., 2014; Aarsland et al., 2017; Bondi et al., 2017). In worst cases, it may impair the autonomic nervous system, which may cause a reduction of somatic and smooth muscle function that leads to a need for consistent care and assistance in their daily routine (Nijssen et al., 2017; Miller et al., 2021).

Oxidative stress and generation of free radicals are few reported causative mechanisms of neurodegenerative diseases in AD (Yan et al., 1994; Christen, 2000), PD (Wei et al., 2018), ALS (Bond et al., 2018) and HD (Jędrak et al., 2018). There is enough evidence to support that oxidative stress has contributed to NDs’ pathogenesis (Lin and Beal, 2006; Bhattacharjee and Borah, 2016).

Reactive oxygen species (ROS) in the forms of hydrogen peroxide (H_2_O_2_), hydroxyl radical (·OH) and superoxide anion radical (O_2_) are generated in various metabolic reactions (Yang and Lian, 2020). They are key regulators of several cellular pathways such as apoptosis, transcriptional regulation, and differentiation (Zhang et al., 2020). However, these free radicals are highly reactive and cause harmful effects when they are unstable. Due to the imbalance of ROS and the antioxidant system at the cellular level, oxidative stress promotes neuronal degeneration and inflammation by damaging vital organic molecules such as protein, lipids, RNA, and DNA (Sarangarajan et al., 2017; He et al., 2020).

Some snake venom components have been reported to exert neuroprotective activity in animal and cellular models (Armugam et al., 2009; Liu et al., 2018; Wang et al., 2018). Venom phospholipase A_2_ is one of the components that have been reported to have neuroprotective activity (Armugam et al., 2009; Wang et al., 2018). There is limited information on the neuroprotective effect of Malaysian snake venom components, including phospholipase A_2_. Therefore, the present study aimed to determine the neuroprotective potential of A2-EPTX-NSm1a against H_2_O_2_-induced oxidative stress-associated neurodegeneration in human SH-SY5Y neuroblastoma cells.

## Materials and methods

### Materials

All chemical reagents used in the present study were analytical grades. Items purchased for isolation and purification of secretory phospholipase A_2_ are stated in a previous study (Abdullah et al., 2021). Culture medium and fetal bovine serum were purchased from Gibco^®^ by Life Technologies™, Massachusetts, US. Accutase Cell Detachment Solution was purchased from Capricorn Scientific GmBH, Ebsdorfergrund, Germany. Retinoic acid was purchased from Sigma-Aldrich, Bornem, Belgium. Human BDNF was purchased from StemCell™ Technologies, British Columbia, Canada. Cell culture plates were purchased from NEST^®^ Biotechnology Co., Ltd., Rahway, US.

### Protein quantification by bicinchoninic acid assay (BCA)

Protein quantification of A2-EPTX-NSm1a using Pierce™ BCA Protein Assay Kit (ThermoFischer Scientific, Massachusetts, US) followed the manufacturer’s protocol. *N. sumatrana* venom and A2-EPTX-NSm1a were diluted in Milli-Q, and bovine serum albumin was used as a standard. The absorbance was measured at 562 nm using a microplate spectrometer (BioTek™ EON™ Microplate Spectrometer, Vermont, US).

### Cell culture and maintenance

Human neuroblastoma cell line SH-SY5Y (ATCC CRL-2266) was seeded at a density of 20,000 cell/cm^2^ on the plate and cultured in Dulbecco Modified Essential Medium (DMEM) High Glucose, GlutaMAX™ supplement, pyruvate (Catalogue No: 10569-010, Gibco^®^ by Life Technologies™, Massachusetts, US) supplemented with 10% heat-inactivated fetal bovine serum (FBS) (Catalogue No: 10270-098, Gibco^®^ by Life Technologies™, Massachusetts, US), Antibiotic-Antimycotic (Catalogue No: 15240-062, Gibco^®^ by Life Technologies™, Massachusetts, US) in humidified 5% CO_2_ and 37°C incubator. The culture medium was replaced every 2 days until the culture reached confluency (70–80%) for sub-culturing or performing assay using undifferentiated cells. Cells were detached using Accutase Cell Detachment Solution (Catalogue No: ACC-1B, Capricorn Scientific GmbH, Ebsdorfergrund, Germany). Cells were cryopreserved below passage 15 to avoid senescence.

### Cell lysate collection

In manufacturing protocol, cell lysates were collected based on the lyse-suspension-cultured mammalian cells technique. Briefly, RIPA lysis and Extraction buffer (Catalogue No: 89900 and 89901, ThermoFischer Scientific, Illinois, US) with the addition of Halt™ Protease and Phosphatase Inhibitor Cocktail (Catalogue No: 78440, ThermoFischer Scientific, Illinois, US) were mixed immediately prior used. Cells were detached and collected by centrifugation at 2,500 g for 5 min. After supernatant were discarded, RIPA buffer was added to the cell pellet and suspended up and down using pipette. The mixture was shaken gently for 15 min on ice and cell debris were separated by centrifuging the mixture at ∼ 14, 000 × g for 15 min. The supernatant was transferred to a new tube and used for experimental.

### Differentiation of SH-SY5Y to cholinergic neurons

Differentiation of SH-SY5Y was performed following Forster et al., 2016 with some modification (Forster et al., 2016). Differentiation of SH-SY5Y was performed in two phases. Phase 1 of the differentiation was maintained with a neurogenic medium with Dulbecco Modified Essential Medium (DMEM) High Glucose, GlutaMAX™ supplement, pyruvate (Catalogue No: 10569-010, Gibco^®^ by Life Technologies™, Massachusetts, US) supplemented with 10 µM retinoic acid (Catalogue No: R2625, Sigma-Aldrich, Bornem, Belgium), 10% FBS and antibiotic-antimycotic (Catalogue No: 15240-062, Gibco^®^ by Life Technologies™, Massachusetts, US) for 4 days. In the second phase, cells were allowed to differentiate in phase 2 medium containing Neurobasal™ Plus medium (Catalogue No: A3582901, Gibco^®^ by Life Technologies™, New York, US), B-27™ Plus Supplement (Catalogue No: A3582801, Gibco^®^ by Life Technologies™, New York, US), 50 ng/ml human recombinant BDNF (Catalogue No: 78005, StemCell™ Technologies, Vancouver, BC, Canada), 10 ng/ml recombinant human FGF-basic (Catalogue No: PHG0266, Gibco^®^ by Life Technologies™, California, US), 20 ng/ml human recombinant EGF (Catalogue No: PHG0311, Gibco^®^ by Life Technologies™, New York, US), 1 µm Dibutyryl-cAMP (Catalogue No: 73882, StemCell™ Technologies, China), 5% heat-inactivated fetal bovine serum (FBS) (Catalogue No: 10270-098, Gibco^®^ by Life Technologies™, Massachusetts, US), antibiotic-antimycotic (Catalogue No: 15240-062, Gibco^®^ by Life Technologies™, Massachusetts, US) until elongated neurite were observed.

### Cell proliferation evaluation

Cell proliferation of differentiated was measured based on the number of cells counted using tryptan blue. Briefly, SH-SY5Y (ATCC CRL-2266) were seeded at a density 10,000 cell/cm^2^ in 6 well plates NEST^®^ Biotechnology Co., Ltd., Rahway, US and allowed to grow for 4 days. Differentiation of SH-SY5Y was initiated with the addition of phase 1 media after the cell growth (Day 0) for 4 days followed with incubation with phase 2 media for the following days until day 14. Media for cell maintenance, cell differentiation in phase 1 and phase 2 were changed in every 2 days. The number of cells was quantified in day 0, 4, 8, 12, and 14. SH-SHSY in neurogenic media 1 containing DMEM, 5% FBS, antibiotic-antimycotic and 10 µm retinoic acid were used as the positive control. Mean data ±SD are shown from three independent experiments.

### Morphology and its neuronal outgrowth

Differentiated SH-SY5Y were allowed to grow in 6 well plates. Cell images were collected every 2 days for 8 days using an inverted microscope (CKX41 Inverted Microscope, Model: CKX41SF, Olympus Life Science, Massachusetts, US). Differentiation of SH-SY5Y was measured based on its neurite length of branched from SH-SY5Y using ImageJ software. Controls were carried out using undifferentiated SH-SY5Y in growth media. Data is expressed as Mean ± SD from three independent experiments.

### Cholinergic neurons confirmation

Characteristics of differentiated SH-SY5Y to cholinergic cells were evaluated based amount of acetylcholinesterase (AChE) using Ellman’s assay with some modification (Ellman et al., 1961). Briefly, 20 µL of cell lysates containing 50 µg of protein, AChE standards (Catalogue No: C2888, SIGMA-Aldrich, Darmstadt, Germany) with concentrations ranging up to 25 mU/ml and 0.1 M phosphate buffer, pH 7.8 as the blank were prepared and added in 96 well plates. Each well was dissolved with 160 µL of phosphate buffer followed by the addition of 10 µL of 3 mm 5,5′-Dithiobis (2-nitrobenzoic acid) (Catalogue No: D218200, SIGMA-Aldrich, Darmstadt, Germany). After that, 10 µL of substrate, 15 mm Acetylthiocholine iodide (Catalogue No: 01480, SIGMA-Aldrich, Darmstadt, Germany) were added and the activity of AChE were measured at 412 nm using a microplate spectrometer (BioTek™ EON™ Microplate Spectrometer, Vermont, US). AChE activity was estimated based on the OD value for every 5 min within 15 min. AChE standard with concentrations 0–25 mU/ml were used to estimate the amount of AChE in the cell lysates. Differentiation of SH-SY5Y was compared with undifferentiated SH-SY5Y, which was used as the control.

### Cell viability

Cells were seeded at density 10,000 cells/cm^2^ in 24 well plates (NEST^®^ Biotechnology Co., Ltd., Rahway, US) and allowed to differentiate until day 8. Cells with 70–80% confluency were treated with A2-EPTX-NSm1a (0–15.265 μg/ml) for 24, 48, and 72 h. The concentration range used in this study was based on a previous cytotoxicity assay of A2-EPTX-NSm1a (Abdullah et al., 2021). MTT solution with final concentration of 0.05 mg/ml was added to each well, and the cells were further incubated for 4 h at 37°C and 5% CO_2_. The insoluble formazan which resulted from oxidation of added MTT by vital cells, was dissolved by addition of 0.1 ml of DMSO and the absorbance of formazan was determined using a plate reader EON microplate spectrophotometer (BioTek™ EON™ Microplate Spectrometer, Vermont, US) at 570 nm. The relative viability of the cells was determined as ratio of optical density of formazan produced by cells treated with A2-EPTX-NSm1a to optical density produced by control cells. For each experiment, the optical density of control cells was considered as 100% of viable cells.

### Neuroprotection assay

Bio-guided assay for neuroprotection analysis for *N. sumatrana* venom and fractions in undifferentiated SH-SY5Y was performed (unpublished data). From the bioguided assay, A2-EPTX-NSm1a showed potential as neuroprotectant in undifferentiated SH-SY5Y (Supplementary material) and thus, neuroprotection assay was performed in the differentiated SH-SY5Y. After differentiation, differentiated SH-SY5Y were treated with H_2_O_2_ for 24 h and the cell viability was evaluated using MTT assay. The concentration of H_2_O_2_ with 40–50% cell viability to the differentiated SH-SY5Y was used in the neuroprotection assay. To analyze the neuroprotection effect of A2-EPTX-NSm1a, differentiated SH-SY5Y was pre-treated with A2-EPTX-NSm1a for 4 h followed by exposure of H_2_O_2_ for 24 h. The neuroprotective effect of A2-EPTX-NSm1a was analyzed based on the cell viability described previously. Groups used for following the assays in understanding the mechanistic of A2-EPTX-NSm1a in protecting cells from oxidative damage were as follows: group 1 was cells with the vehicle (control), group 2 was cells with H_2_O_2_ (oxidative stress group), group 3 was cells with pre-treated of 0.977 μg/ml A2-EPTX-NSm1a for 4 h followed by addition of H_2_O_2,_ group 4 was cells with 0.977 μg/ml A2-EPTX-NSm1a.

### Anti-apoptosis analysis by caspase assays

Cells were grown and differentiated in T75 flask until the cells reached 70–80% confluency. Cells were treated with A2-EPTX-NSm1a for 4 h followed by exposure of H_2_O_2_ for the next 20 h. Cells without the addition of A2-EPTX-NSm1a and H_2_O_2_ were used as the control group. To assess the effect of A2-EPTX-NSm1a on H_2_O_2_-induced apoptosis in differentiated SH-SY5Y cells, the caspase activity for caspase-3 and caspase-8 were measured by colorimetric assay. Cells were lysed with a lysis buffer and caspase activities were performed as mentioned in the manufacturer protocol (Raybio^®^ Caspase-3/Caspase 8 Colorimetric Assay Kit, Catalogue No: 68CL-Casp3/68CL-Casp8, RayBiotech, Inc, Georgia, US). Briefly, 50 µg of lysed protein was used in this study and diluted with a diluent solution. DEVD-*p*NA and IETD-pNA are the substrate used for the caspase 3 and 8 activity, respectively. The mixtures were incubated in 37°C for 2 h and absorbance was read at 405 nm. The relative activity of caspases in the treated group were compared with the control.

### Electron microscopy

Cells at the density of 10, 000 cells/cm^2^ in 6 well plates were grown and differentiated until it reached 70–80% of confluency. For the electron microscopy analysis, 1 ml of sediment sample from the cell culture grown with A2-EPTX-NSm1a prior addition of H_2_O_2_, H_2_O_2,_ A2-EPTX-NSm1a and growth media as the control was mixed with 2.0% glutaraldehyde in 0.1 N PBS for fixation process. After fixation minimum 1 h, glutaraldehyde was discarded, and the sample was washed before proceeding with post fixation using 1% osmium tetroxide for 1 h until the pellet turned black. The culture cells were exposed to *en-bloc* staining by using uranyl acetate zero for 5 min. After that, the sample will go through a dehydration process using acetone in graded series starting at 70%, 90%, and 100% (three times, 3x) concentration. In transition between dehydration and embedding with epoxy resin, the sample was infiltrated with 1: 1 (resin:100% acetone) mixture epoxy. Embedded samples were polymerized at 60°C overnight and then, 90 nm thin-sectioned using an ultramicrotome UC7 (USA) model were carried out and transferred to copper 150-mesh electron microscopy grids. The grid is viewed under TEM FEI, Tecnai Spirit G2. Total number of cells studied were at least 30 cells from 8 layers of slices of each treatment groups. Random images of mitochondria were captured to prevent bias. The number and length of mitochondria were measured using ImageJ.

### Human neuro array

Cells were treated as described for anti-apoptosis evaluation and cell lysates were collected as described in the previous section. The effect of neuronal brain markers was evaluated using G-series Human Neuro Discovery Array 1 (Catalogue No: GSH-NEU-1, RayBiotech, Inc, Georgia, US) followed the manufacturing protocol. Briefly, the glass slides were completely air-dried before wells in each slide were blocked with sample diluent for 30 min at room temperature. 500 μg/ml of protein for cell lysates were added in each well after the diluent solution were decant and incubated for overnight at 4°C. Well in each step were then followed the protocol with several steps of washing, and incubation with the biotinylated antibody cocktail and Cy3 equivalent dye-streptavidin. The signal intensity of different cytokines on the slide were scanned using Agilent DNA Microarray Scanner (C series) and the data from the slides were extracted using a software, Agilent Feature Extraction, version 12.1.0.3. Data were automatically normalized, and mean data were presented based on the signals in arbitural unit (AU).

### Statistical analysis

All data analyses were performed using GraphPad Prism 9.0 software (GraphPad Software Inc, La Jolla, Canada). Data expressed as mean ± SEM of three independent experiments (*n* = 3) in triplicate. The significant level of A2-EPTX-NSm1a between groups was determined *via t*-test or one-way variance (ANOVA) followed by Tukey’s post-test. Values of *p < 0.05* were considered significant.

## Results

### Differentiation of SH-SY5H to cholinergic neurons

**FIGURE 1:**
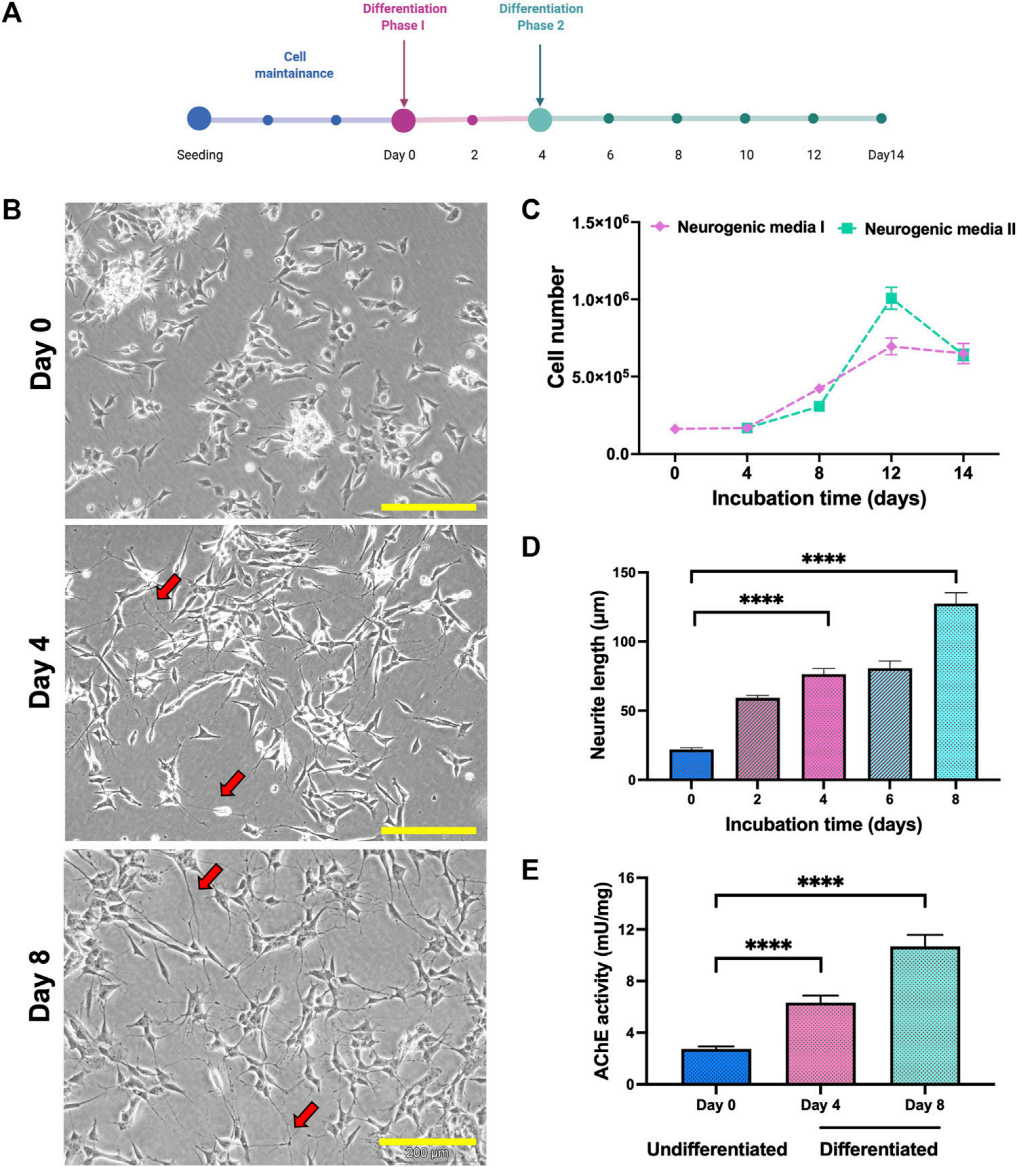
Differentiation of SH-SY5Y cells. **(A)** SH-SY5Y were differentiated as in the line chart. **(B)** Phase contrast of SH-SY5Y cells morphology on days 0, 4 and 8. Neurite extension labelled in red arrow. Scale bar = 200 μM. **(C)** Differentiation of SH-SY5Y showed modification in the cell number, **(D)** neurite length and **(E)** AChE activity. Data were presented as means ± SD, where *p* < 0.05 is considered significant. **** indicated *p* < 0.001.

SH-SY5Y cells were differentiate to cholinergic neurons up to 14 days in two different phases (Figure 1A). Differentiation of SH-SY5Y was initiated with 5% FBS and 10 µm retinoic acid (RA) in phase 1. In phase 2, differentiation continued following the addition of other differentiation factors, which were human recombinant Brain-Derived Neurotrophic Factor (BDNF), Epidermal growth factor (EGF), Fibroblast growth factor (FbF) and dibutyril cyclic adenosine monophosphate (dcAMP). From the morphological analysis, the cells showed extended neurites outgrowth compared to the undifferentiated cells in both the phases (Figure 1B). The number of cells were elevated at day 8, where medium with RA was used as the positive control (Figure 1C). The length of neurite outgrowth was measured, and cells in phase 2 containing differentiation factors showed longer neurite length compared to the undifferentiated cells on day 0 (Figure 1D). The characteristic of differentiated SH-SY5Y was characterized based on the AChE activity in the cell lysate, and the AChE activity was elevated in differentiation method. Based on these criteria, differentiation of SH-SY5Y was continued until day 8 and these cells were used for neuroprotection study.

### Confirmation of cholinergic population

Cholinergic population of differentiated cells were confirmed by elevation of acetylcholine esterase (AChE) activity in the cell lysate. The introduction of neurogenic media in both phases significantly increased the AChE activity and differentiated cells (day 8) showed higher AChE activity levels compared to the undifferentiated cells (Figure 1E).

### A2-EPTX-NSm1a protects SH-SY5Y from H_2_O_2_ induced cytotoxicity

**FIGURE 2:**
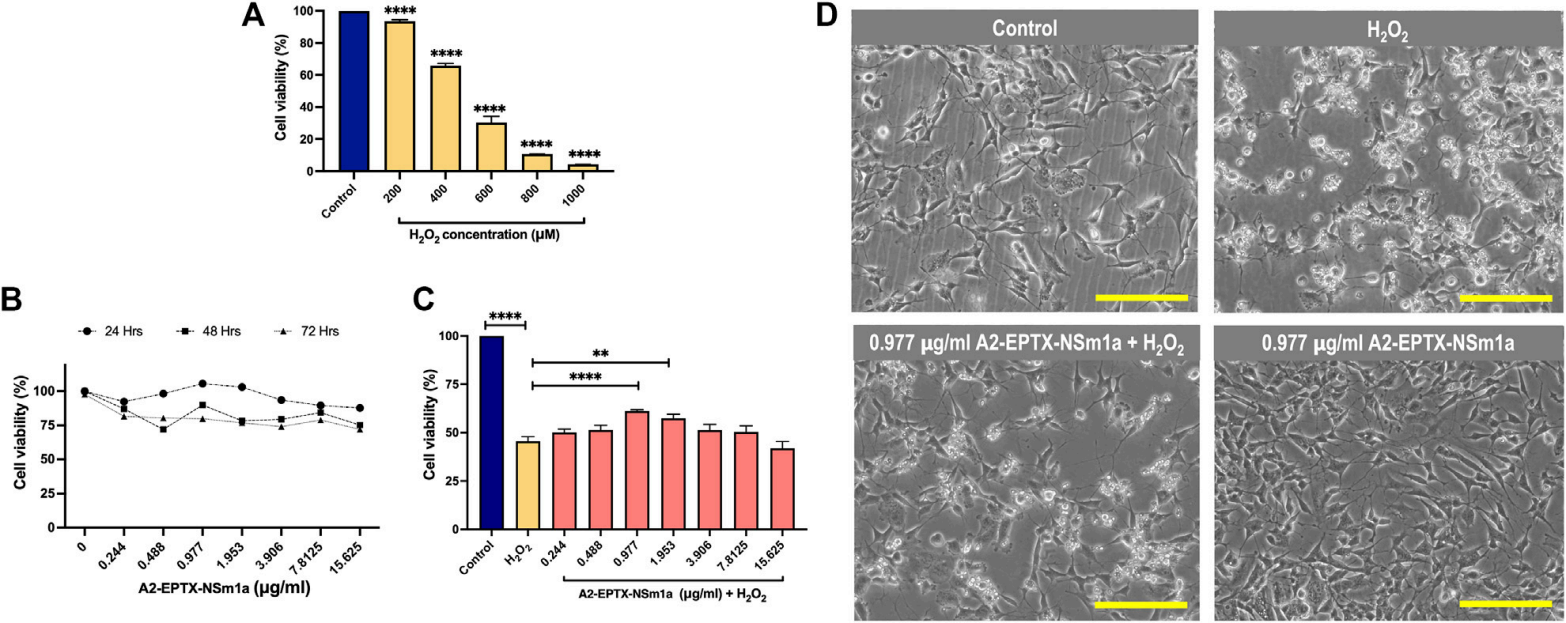
Cell proliferation and neuroprotection of a secretory phospholipase A2, A2-EPTX-NSm1a in differentiated SH-SY5Y cell lines. **(A)** Cytotoxicity of H_2_O_2_ on differentiated SH-SY5Y cells. **(B)** Cell proliferation of A2-EPTXNSm1a was evaluated at different times of incubation. **(C)** Neuroprotection assay of A2-EPTX-NSm1a. **(D)** Phase contrast of differentiated SH-SY5Y in the different groups - untreated (control), H_2_O_2_ only (oxidative group), pre-treatment of 0.977 µg/ml A2-EPTX-NSm1a followed by H_2_O_2_ and 0.977 µg/ml A2-EPTX-NSm1a only after 24 h of incubation time. Scale bar = 200 μM.

Cytotoxicity of H_2_O_2_ in differentiated SH-SY5Y indicated viability of cells were reduced for 50% at 450 µm (Figure 2A). Cell proliferation of A2-EPTX-NSm1a at concentrations 0.244 μg/ml to 15.625 μg/ml is more viable in incubation for 24 h as compared to 48 h and 72 h (Figure 2B). Based on the neuroprotection assay, pre-treatment A2-EPTX-NSm1a at a concentration of 0.977 μg/ml significantly modified the cell viability of cells from 45.6 ± 7.0% to 61.1 ± 1.8% from cell death induction by H_2_O_2_ (Figures 2C,D).

### A2-EPTX-NSm1a reduced caspases activities

**FIGURE 3:**
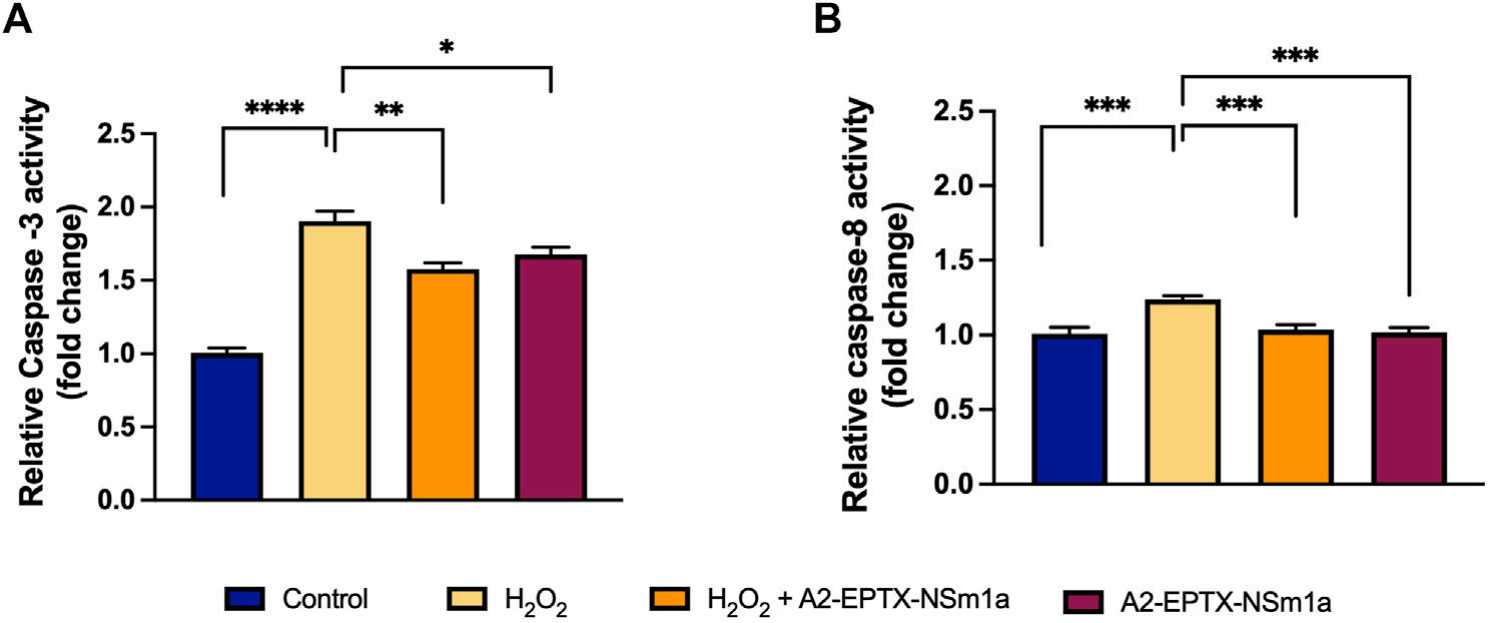
Effect of pre-challenged A2-EPTX-NSm1a on caspase-3 and caspase-8 activity in differentiated SH-SY5Y with H_2_O_2_. **(A)** Relative Caspase-3 activity (fold change); **(B)** Relative Caspase-8 activity (fold change) All experiment was conducted in triplicate (*n* = 3). Data are presented as the mean ± SD. * *p* < 0.05, ** *p* < 0.01, *** *p* < 0.005, **** *p* < 0.001 as compared to the H_2_O_2_-treated group.

The apoptosis activity of A2-EPTX-NSm1a was assessed on the level of caspase-3 and caspase-8 activity in the cells (Figure 3). H_2_O_2_ significantly increased the caspase-3 level and caspase-8 (Figures 3A,B) compared with the control group. Pre-treatment using A2-EPTX-NSm1a or A2-EPTX-NSm1a alone reduced caspase-3 level (Figure 3A). Caspase-8 level was slightly increased in the treatment of H_2_O_2_ but pre-treatment of A2-EPTX-NSm1a and A2-EPTX-NSm1a alone did not cause a significant increase in the caspase-8 activity level compared to the control (Figure 3B).

### A2-EPTX-NSm1a protects from cellular apoptosis

**FIGURE 4:**
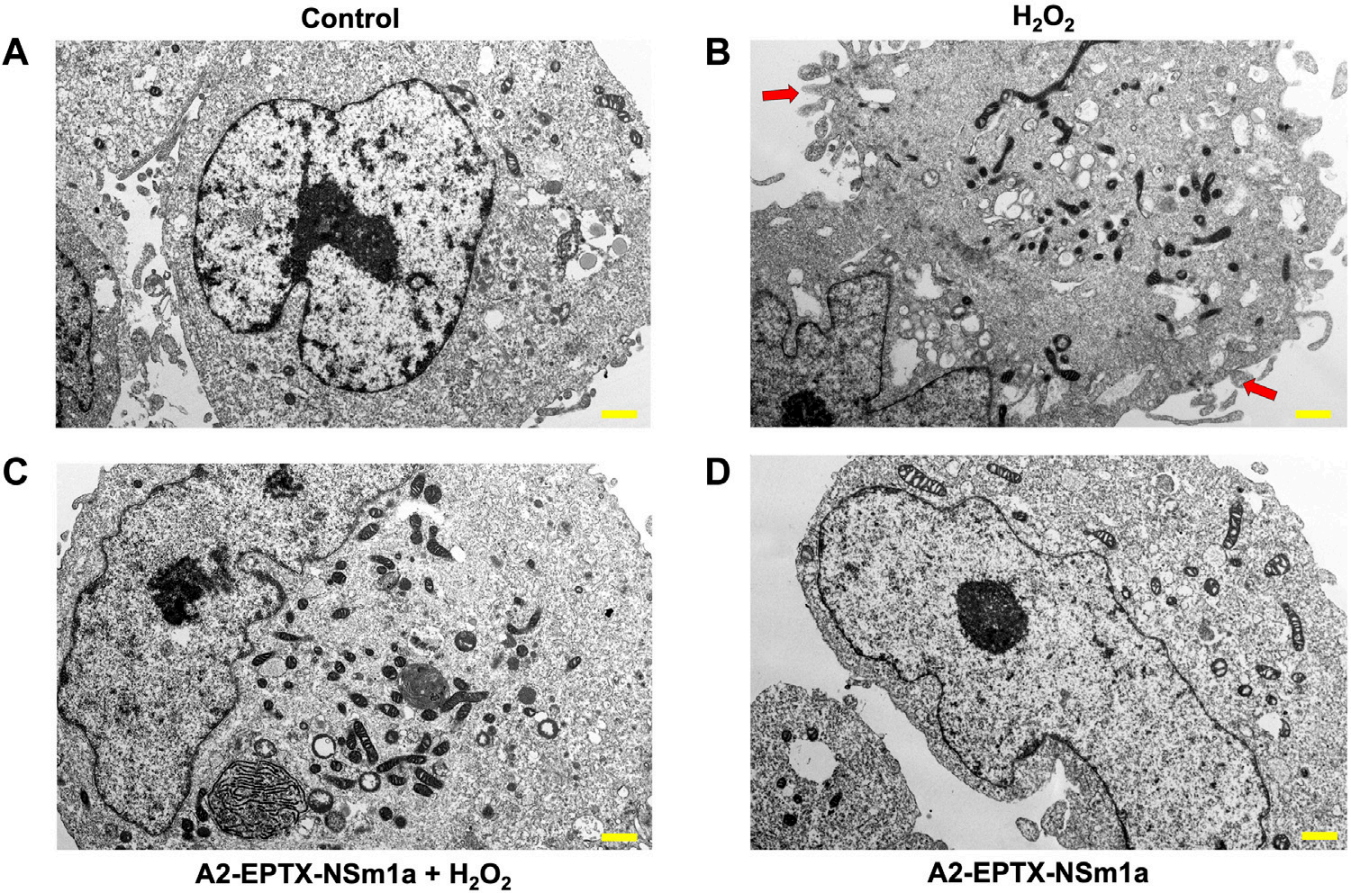
Representative cell structure images of differentiated SH-SY5Y observed in an electron microscope. Image of differentiated SH-SY5Y cells in **(A)** untreated group (Control), **(B)** H_2_O_2_-induced neurotoxicity group, **(C)** addition of pre-treatment of A2-EPTX-NSm1a following the addition of H_2_O_2_ and **(D)** A2-EPTX-NSm1a alone at 24 h of incubation. Scale bar = 1 μm. Arrow showed the formation of membrane bubbling due to cell apoptosis induced by H_2_O_2_.

Cell apoptosis was evaluated by altering cell morphology viewed under an electron microscope (Figure 4). Membrane plasma of cells was not disrupted in control (untreated cells) (Figure 4A). However, cell membrane with incubation of H_2_O_2_ for 24 h showed formation of babbling membrane and irregular shape of nucleus (Figure 4B). Cells with pre-treatment of A2-EPTX-NSm1a showed normal membrane structure with fewer bubbling membrane structure and less irregular shape (Figures 4C,D).

### A2-EPTX-NSm1a altered number and length of mitochondria

**FIGURE 5:**
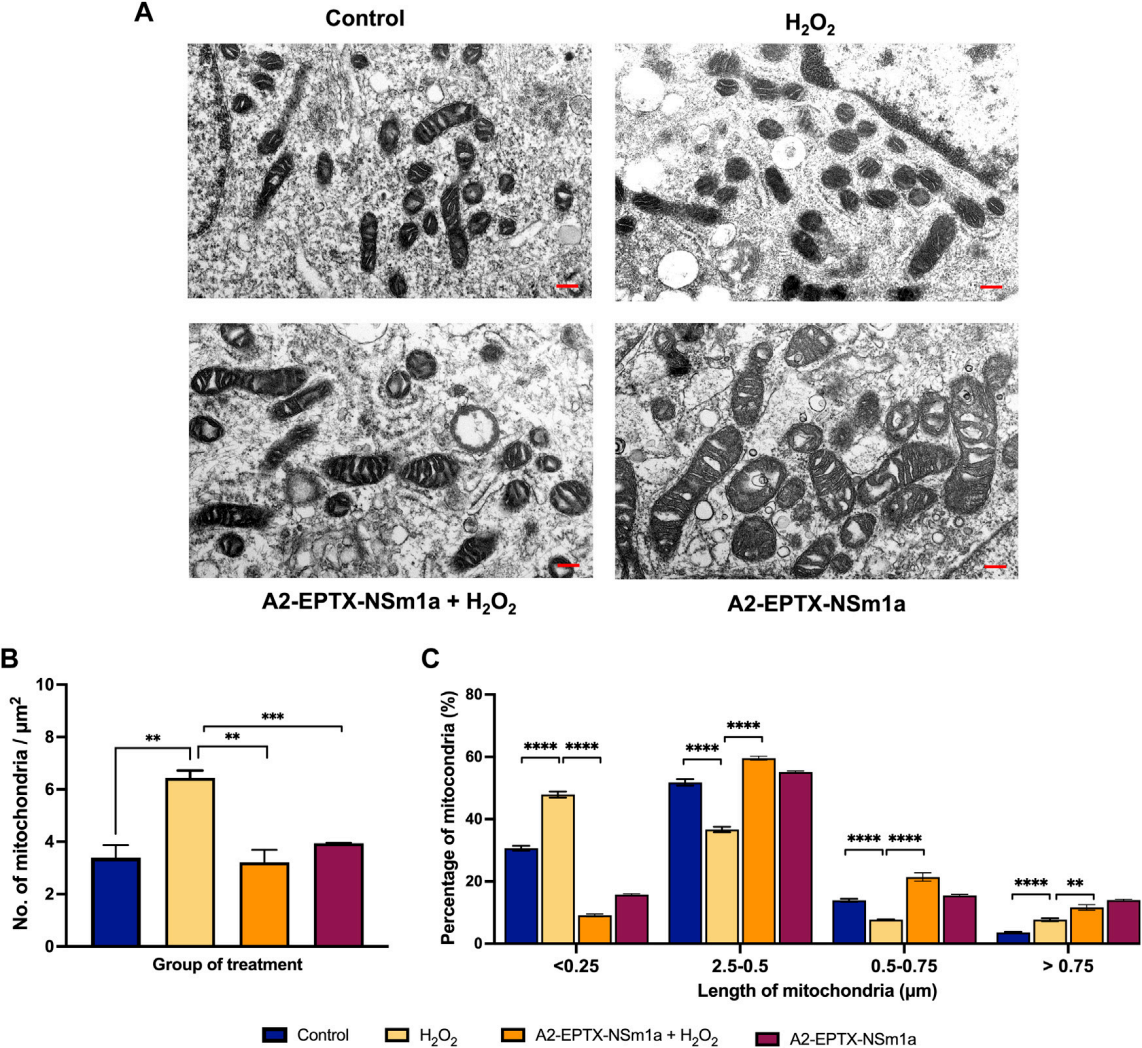
Observation of mitochondrial morphology from electron microscope. **(A)** Morphology of mitochondria in each group observed after 24 h of treatment in a representative of cell. **(B)** Number of mitochondria in the area has been quantified and the length of mitochondria in each group were measured using ImageJ analysis. Scale bar = 200 nm. Data showed mean ± SEM and marked with ** indicating *p* < 0.01, *** as *p* < 0.005, and **** marked as *p* < 0.001.

The effects of A2-EPTX-NSm1a in protecting cell death was further evaluated *via* quantification of mitochondria in the cells (Figure 5A). The morphology observation revealed the different sizes of mitochondria. Quantification of the number of mitochondria per area with the introduction of H_2_O_2_ in cells, increased the numbers of mitochondria as compared to the control (Figure 5B). Pre-treatment with A2-EPTX-NSm1a attenuated the number of mitochondria in cells with H_2_O_2_ (Figure 5B). This pattern was also similar to the effect of A2-EPTX-NSm1a alone (Figure 5B). Further evaluation on the size of the mitochondria indicated 47.9% of mitochondria treated with H_2_O_2_ were in length below 0.25 µm. (Figure 5C). Interestingly, the number of mitochondria in this length was reduced in cells treated with A2-EPTX-NSm1a, with or without H_2_O_2_ induction (Figure 5C). Interestingly, we also identified that A2-EPTX-NSm1a expands the length of mitochondria as compared to control and H_2_O_2_ alone.

### A2-EPTX-NSm1a reduced neuroinflammation

**FIGURE 6:**
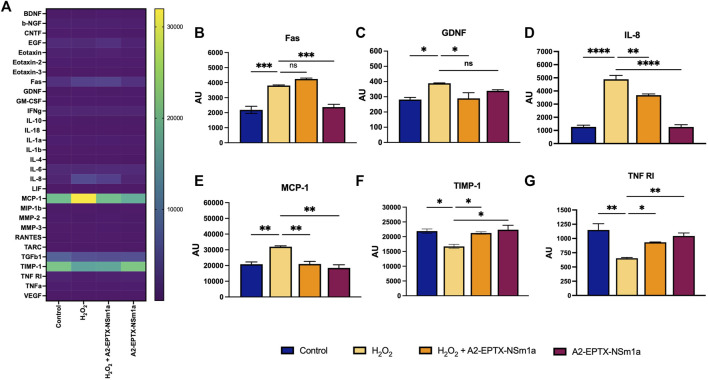
A2-EPTX-NSma1 effects on the neuro-markers expression levels in the differentiated SH-SY5Y with and without oxidative stress conditions. The expression level of proteins was analysed using normalized signals extracted from Agilent Feature Extraction software. Values are collected from quadruplicates of a specific protein and expressed as mean ± standard deviation (*n* = 3). Protein expression is considered as significant as compared to the H_2_O_2_ group with *p* < 0.05 based on statistical analysis using one-way ANOVA followed by Tukey’s post-test. ns as significant, * *p* < 0.05, ** *p* < 0.01, *** *p* < 0.005, **** *p* < 0.0001.

The protein array study showed modification of the expression of multiple neuronal markers after 24 h of different treatment groups (Figure 6A). Interestingly, among 30 markers, the expression of six protein markers was significantly altered (Figures 6B–D). Protein expression of GDNF, IL-8, and MCP-1 were reduced in the cells pre-treated with A2-EPTX-NSm1a prior to H_2_O_2_ induction (Figures 6C–E). In contrast, TIMP-1 and TNF RI were upregulated with the presence of A2-EPTX-NSma1a compared to H_2_O_2_ alone (Figures 6B,F). No significant changes of A2-EPTX-NSm1a with H_2_O_2_ induction on FAS expression compared to H_2_O_2_ alone, but Fas level of A2-EPTX-NSm1a with H_2_O_2_ induction and H_2_O_2_ were higher than control (Figure 6B).

## Discussion

This study determined the potential neuroprotective activity of a PLA_2_ from Malaysian *N. sumatrana* venom, A2-EPTX-NSm1a, using *in-vitro* experiments. Our previous study found that A2-EPTX-NSm1a was less cytotoxic in SH-SY5Y as compared to *N. sumatrana* crude venom (Abdullah et al., 2021), suggesting A2-EPTX-NSm1a may exert other activities in neuronal cells.

To further understand the role of A2-EPTX-NSm1a in protecting neuronal cells, A2-EPTX-NSm1a was assessed in differentiated SH-SY5Y under oxidative stress conditions. Differentiated SH-SY5Y cells were found to display neuronal characteristics with the addition of RA and BDNF. These results were very much in-line with the previously reported studies, where an additional 10 µm RA inhibits the proliferation of SH-SY5Y (Påhlman et al., 1984; Encinas et al., 2000). Treatment with retinoic acid resulted in cells that acquired neuron-like phenotype increased homogenous cell population and expressed tyrosine kinase receptor B (TrkB) on the surface of the cells after 3 days of incubation (Encinas et al., 2000). The introduction of BDNF, an agonist of the TrKB receptor, continues to expand the neurite length of cells, increase d the connection between cells, and thus, increase the cells survival (Encinas et al., 2000; Dravid et al., 2021). Other than RA and BDNF, we added dcAMP, EGF and FGF to maintain the cell growth and neurite outgrowth of SH-SY5Y to mature neuron cells (Even et al., 2009; Pirou et al., 2017; Huang et al., 2021) (Figures 1B–D). Similarly, other studies also support that the addition of these growth factors has been applied in human iPSC to characterize differentiation to neural progenitor cells (Kang et al., 2017). Further evaluation of the endogenous acetylcholinesterase assay has demonstrated an elevation of enzyme activity indicating cholinergic rich cultures, which is suitable for the cholinergic system and is a suitable target for AD *in vitro* model (Ferreira-Vieira et al., 2016; Wang et al., 2016). Therefore, with our findings, we successfully differentiated SH-SY5Y to the matured cholinergic neurons, which was used further in the study.

Oxidative stress is caused by the generation of reactive oxygen species (ROS) such as H_2_O_2_ produced by metabolism in the biological system. ROS plays a vital role mainly as a signaling agent, but excessive ROS due to imbalance of ROS generation and ROS elimination also with lack of antioxidants disturb the biochemical process in the cell. This is because ROS is capable of oxidizing all biomolecules, including protein, lipid, and nucleic acid (Cross et al., 1987). Abnormal oxidation caused by ROS leads to oxidative stress, which has been associated with the progression of pathogenesis for many diseases, including neurodegenerative disease (Singh et al., 2019). In the cellular level, brain neuron cells use high consumption of oxygen for metabolism. In addition, the brain is rich in polyunsaturated fatty acid and presence of low level of antioxidants, thus, making brain cells vulnerable to oxidative stress which leads to activation of programmed cell death known as apoptosis. Hence, oxidative stress has been reported to have a strong link with the progress of pathogenesis of many neurodegenerative diseases.

In the present study, H_2_O_2_ reduced the cell viability of differentiated SH-SY5Y in a concentration-dependent manner when tested at varied increasing concentrations starting from 200–1,000 µm for 24 h incubation (Figure 2A). Interestingly, secretory PLA_2_ from *Naja sumatrana* venom (named A2-EPTX-NSm1a) maintained cell viability of differentiated SH-SY5Y cells (Figure 2B). This could be due to its characteristic that have less toxicity to SH-SY5Y than the crude venom as previously reported (Abdullah et al., 2021). We further evaluated the potential of PLA_2_ compound as a neuroprotectant against H_2_O_2_. Pre-treatment with A2-EPTX-NSm1a 4 h prior to H_2_O_2_ challenge, protects the differentiated SH-SY5Y cells from oxidative stress damage. Additionally, secretory PLA_2_ purified from *N. sputatrix* venom was also reported to have a neuroprotective effect in rat stroke models (Armugam et al., 2009).

It is well established that an exposure of extensive ROS activates the caspase cascade. In the present study, H_2_O_2_ elevated the activity of both caspase-3 and caspase-8. However, the activity of caspase-3 is highly significant compared to caspase-8 when H_2_O_2_ was added to the cultures. Modulation in the caspase-3 and caspase-8 activities indicates the activation of caspase pathways. Activation of caspase-3 activates the intrinsic pathway of apoptosis and caspase -8 is related to extrinsic apoptosis pathway. These results suggested that A2-EPTX-NSm1a is effective against the oxidative stress damage of cholinergic neurons by H_2_O_2_. During the apoptosis process, the morphology of cells gets modified due to changes in the biochemical property of cells and eventually causes damage to the cells. Apoptosis causes cells to shrink and undergo blebbing, which was evident from microscopical analysis when H_2_O_2_ alone was introduced to the cells. In our study, pre-treatment of A2-EPTX-NSm1a reduced the activity of caspase-3 and normalized the caspase-8 activity. Furthermore, as observed under an electron microscope, A2-EPTX-NSm1a protects the cell membrane disruption where less membrane blebbing was observed.

In AD and other neurodegenerative diseases, protein misfolding compromises the energy supply and antioxidant system, leading to synaptic and mitochondrial dysfunction. As Mitochondria is an important organelle involved in ATP production, and it has been reported to play an important role in pathogenesis of neurodegenerative diseases such as PD (Nicoletti et al., 2021), AD (Misrani et al., 2021) and Huntington’s disease (Intihar et al., 2019). Mitochondria size, shape, number and location can be naturally altered as part of maintaining the biology and quality of mitochondria (Fu et al., 2019). The process is known as mitochondrial dynamics where two unique processes are involved, fission (division) and fusion. They are important for cell viability and synaptic activity. Therefore, perturbations in the mitochondria dynamic lead to neuronal defects (Reddy and Oliver, 2019).

In our study, the number of mitochondria was higher compared to the control in oxidative stress condition (Figure 5B). This could be due to mitochondria fragmentation with the introduction of the oxidative stress agent, H_2_O_2_ which was previously reported (Fan et al., 2010; Garcia et al., 2018). Interestingly, pre-treatment of A2-EPTX-NSm1a with H_2_O_2_ reduced the number of mitochondria similarly found in A2-EPTX-NSm1a alone (Figure 5B). As we further investigated on the length of mitochondria, our study showed the number of mitochondria at length below 0.25 µm is increased under oxidative conditions (Figure 5C). In contrast, mitochondria of bigger size were increased with the addition of A2-EPTX-NSm1a, similarly found in control. Oxidative stress triggered the mitochondria fragmentation (Wu et al., 2011) in cells. Thus, we suggest that A2-EPTX-NSm1a protects the viability of cells from oxidative damage *via* reduction of mitochondria fragmentation. Therefore, to balance the cycle of mitochondria dynamic, mitochondria will naturally be fused and in some condition, hyper-fusion mitochondria happen to allow the cell to survive (Das and Chakrabarti, 2020). In our study, A2-EPTX-NSm1a revealed increased number of mitochondria at larger size (more than 0.5 µm and 0.75 µm), suggesting that A2-EPTX-NSm1a increased the mitochondria fusion and reduced the fission process to avoid mitochondria fragmentation from further process mitophagy in relate to improve mitochondrial function (Das and Chakrabarti, 2020). Our findings indicated A2-EPTX-NSm1a showed neuroprotective activity and showed an indication of anti-apoptosis based on the caspase activity, cell morphology and the mitochondria evaluation. Consistently, reduction of caspase activity and an increased in cell viability have also been observed with the treatment of snake venom phospholipase A_2_ from *N. sputatrix* venom (Armugam et al., 2009).

Oxidative stress modified the molecular mechanism in response to the exposure to ROS (Yang et al., 2019) and declined the neurological function (Wang and Michaelis, 2010). One of the factors associated with the progression of neurodegenerative diseases is the activation and recruitment of microglial in the regions with neuronal damage and death (Boillée et al., 2006). Recruitment of inflammatory cells is due to the release of cytokines and chemokine by the neuronal cells and the sequentially release a variety of inflammatory mediators and trophic factors that contribute to the pathogenesis of the neurodegenerative disease (Flügel et al., 2001). This was proved by the recruitment of microglial and T-cells in a number of brain injury that seems to be attracted by neurons (Troost et al., 1989). One of the factors contributing to the recruitment of inflammatory cells is the release of cytokines and chemokines by the neuronal cells (Flügel et al., 2001) and sequentially secrete a variety of inflammatory mediators and trophic factors, which then contribute to the pathogenesis of neurodegenerative disease. This was proved by the recruitment of microglial and T-cells in a number of brain injury that seems to be attracted by neurons (Troost et al., 1989). Continuous neuroinflammation in the central nervous system activated astrocyte and microglial cells that are responsible for the recovery of neurodegenerative disease such as in AD, PD and frontotemporal dementia (Bachiller et al., 2018).

Oxidative stress and inflammation have been the focus to prevent the incidence of further neurological disorders (Wu et al., 2020). In H_2_O_2_-induced oxidative stress triggered protein expression of several inflammatory mediators, including IL-8, IL-11, TNF-alpha, MCP-1, VEGF, and many other pro-inflammatory markers (Flügel et al., 2001; Baron et al., 2005; Galasko and Montine, 2010) using protein array. Interestingly, most of these proteins were significantly normalized with the addition of A2-EPTX-NSm1a. Under oxidative stress condition, expression of cytokines, MCP-1 and IL-8, the neutrophil/monocyte chemo-attractants, respectively was expressed with the introduction of H_2_O_2_. In parallel to our findings, gene and protein expression of MCP-1 and IL-8 were found to be highly expressed in human monocytic THP-1 cells (Akhter et al., 2021) and other cells (Flügel et al., 2001; White et al., 2005; Kawaguchi-Niida et al., 2013) under oxidative stress conditions. Even though the relation between oxidative stress and cytokines release is not fully understood, however, studied by Akhter et al., 2021, suggested expression of MCP-1 and IL-8 in cells is mediated *via* NF-κB and ERK1/2 signalling (Akhter et al., 2021).

It has been demonstrated that activation of NFκB (nuclear factor kappa B) pathway leads to the production of pro-inflammatory cytokines (Morgan and Liu, 2011). Other than MCP-1 and IL-8, a small protein named GDNF was also found to be highly expressed in response to oxidative stress conditions. In the brain, the reduction of pro-inflammatory markers causes reduction in activation of microglial and brain leukocyte infiltration, which lead to brain edema and further complication. Release of neurotrophins such GDNF strengthens the blood-brain barrier integrity and nurtures the neighbouring neuron. Interestingly, the reduction of MCP-1 and IL-8 in cells with pre-treated with A2-EPTX-NSm1a suggests A2-EPTX-NSm1a reduced inflammation of cells *via* reduction the expression of the pro-inflammatory markers. Consistent with our study, the beneficiary effect of snake venom phospholipase A_2_ in controlling the inflammation has been reported previously from a study with pre-condition of secretory phospholipase A_2_ named sPLA2 derived from *Naja mossambica* that improves neurological disorder in rats (Wang et al., 2018). This study reported that sPLA2 reduced inflammation in the surgical brain injury model *via* multiple mechanisms, including activation of peripheral PLA2-5LOC-LTB4 cascade and neuroinflammation (Wang et al., 2018).

These findings suggested that A2-EPTX-NSm1a protects oxidative stress damage of cells *via* increasing the survivability of cells. Elevation of expression of trophin factor GDNF was detected in cells pre-treated with A2-EPTX-NSm1a. In healthy brain, GDNF is exclusively expressed in neurons and forms a complex with GDNF family receptor α1 (GFRα1). The complex of GDNF/GFRα1signals that interacts through RET “rearranged during transfection” in several pathways in neurons provided pro-survival effects (Duarte Azevedo et al., 2020). Additionally, A2-EPTX-NSm1a was found to increase the expression of TNF R1 in cells that controlled the survival of cells with its cell death domain *via* binding of TNF- *α*. Previous study has shown that the neuronal damage was greater in TNFR-knockout mice compared to the wild-type mice in stroke and epileptic seizure model (Bruce et al., 1996). Additionally, in a study by Lu, et al., neuronal damage, and glial activation in the hippocampus were significantly enhanced in TNFR-knockout mice compared to the wild-type mice in the kainic-acid induced seizure study (Lu et al., 2008), suggesting the protective role of the TNFR-1 signal. Furthermore, we also identified the effect of A2-EPTX-NSm1a on TIMP-1 that responsible for regulating MMPs. In many neuronal injuries, changes in MMPs expression cause more severe injury, such as disrupting the blood-brain barrier, and elevate the incidence of brain hemorrhage and neuroinflammation (Minta et al., 2020). Therefore, the reduction of MMPs activity helps in reducing the abnormal cleavage of ECM, decreased the incident of chronic inflammation and exacerbate of inflammation, inhibits activation of cell death receptor, free radicals and immune cells (Behl et al., 2021). Recently, inhibition of MMPs has been researched as a target to slow the progression of neuronal damage in AD (Zipfel et al., 2020).

## Conclusion

In this study, we have demonstrated that A2-EPTX-NSm1a, a secretory phospholipase A_2_ from *N. sumatrana* venom promotes cell survival under oxidative stress conditions. A2-EPTX-NSm1a protects neuronal from oxidative stress damage through inhibition of cell apoptosis and altering protein expression of markers for neuroinflammation and apoptosis. Therefore, this study provides additional knowledge on the functions and activities of snake venom phospholipase A_2_ in neuronal cell lines and a basis for further research on the activity A2-EPTX-NSm1a in oxidative stress-induced neuron cells. It also opens the possibility of using A2-EPTX-NSm1a or any proteins from *N. sumatrana* venom as a candidate for drug discovery or research tool in oxidative stress-induced neurodegenerative diseases research.

## Data Availability

The raw data supporting the conclusions of this article will be made available by the authors, without undue reservation.
