# Supplementary material for: Neuroprotective effect of phospholipase A2 from Malaysian Naja sumatrana venom against H2O2-induced cell damage and apoptosis
Source: Front Pharmacol. 2022 Oct 14;13:935418. doi: 10.3389/fphar.2022.935418 (PMC9614335; doi:10.3389/fphar.2022.935418)
Supplement: Supplementary file 1 [file DataSheet1.PDF]

Supplementary Material

Neuroprotection of A2-EPTXNSm1a in undifferentiated SH-SY5Y cells

1 Supplementary Methods

1.1 Cell culture maintenance

Undifferentiated SH-SY5Y (ATCC CRL-2266) was used for the bio-guided assay. SH-SY5Y was grown and maintained in Dulbecco Modified Essential Medium (DMEM) High Glucose, GlutaMAX™ supplement, pyruvate (Catalogue No.: 10569-010, Gibco® by Life Technologies™, Massachusetts, US) supplemented with 10% heat-inactivated fetal bovine serum (FBS) (Catalogue No.: 10270-098, Gibco® by Life Technologies™, Massachusetts, US), Antibiotic-Antimycotic (Catalogue No.: 15240-062, Gibco® by Life Technologies™, Massachusetts, US) in humidified 5% CO_2 and 37°C incubator.

1.2 Cell viability

Identification of optimum dosage for H_2O_2 and cell viability of A2-EPTXNSm1a in undifferentiated SHSY5Y was performed in MTT assay using thiazolyl blue tetrazolium bromide (SIGMA M2128). Cells were seeded 10, 000 cell/cm^2 and maintained in medium as described earlier. H_2O_2 with concentration 100, 200, 300, 400 and 500 µM and A2-EPTX-NSm1a were treated at concentration 0.061, 0.122, 0.244, 0.488, 0.977, 1.953 and 3.906 µg/ml were treated in each well with cells growth at 70-80% confluency and incubated for 24 hours at 37°C in 5% CO_2 humidified atmosphere. Selection of A2-EPTXNSm1a concentration were selected based on cell viability with more than 90% in previous finding (Abdullah et al., 2021). At the end of treatment, MTT solution with final concentration 0.05 mg/ml were added to each well and the cells were further incubated for 4 hours at 37°C and 5% CO_2 in humidified atmosphere. The insoluble formazan, which resulted from oxidation of added MTT by vital cells, was dissolved by addition of 0.1 ml of DMSO and the absorbance of formazan was determine using a plate reader EON microplate spectrophotometer (BioTek Instruments, USA) at 570 nm. The relative viability of the cells was determined as ratio of optical density of formazan produced by cells treated with H_2O_2 and A2-EPTX-NSm1a to optical density produced by control cells. For each treatment, the optical density of control group was considered as 100% of viable cells.

1.3 Neuroprotection assay

Neuroprotection assay was performed in 24 wells plates with cells density of 10,000 cells/cm^2 and allowed to grow until it reached 70-80% confluency under the above cell culture maintenance. A2-EPTXNSm1a were pre-treated for 4 hours before addition of H_2O_2. H_2O_2 concentration with 40-50% cell viability to the cells was used in the neuroprotection assay and incubated for 24 hours at 37°C in 5% CO_2 humidified atmosphere. At the end of treatment, MTT solution with final concentration 0.05 mg/ml were added to each well and the cells were further incubated for 4 hours at 37°C and 5% CO_2 in humidified atmosphere. The insoluble formazan, which resulted from oxidation of added MTT by vital cells, was dissolved by addition of 0.1 ml of DMSO and the absorbance of formazan was determine using a plate reader EON microplate spectrophotometer (BioTek Instruments, USA) at 570 nm.

2 Supplementary Results

2.1 Cell viability of H_2O_2

Cell viability of indicated 40 to 50% of cell viability with introduction of 200 to 300 µM H_2O_2. Therefore, H_2O_2 with concentration 250 µM has been used in the neuroprotection assay using undifferentiated SH-SY5Y. Cell viability evaluation indicated cell viability with A2-EPTxNSm1a treatment showed cell viability with more than 80% and the same concentrations of A2-EPTxNSm1a has been used in neuroprotection assay.

2.2 Neuroprotection of A2-EPTxNSm1a on undifferentiated SH-SY5Y

A2-EPTxNSm1a showed neuroprotection against H_2O_2 at 24 hours of incubation on undifferentiated SH-SY5Y cell line. Cell viability of undifferentiated cells in H_2O_2 treatment showed 43.7% and A2-EPTxNSm1a elevated significantly of the cell viability to 60.4% in 0.122 µg/ml, 62.7% in 0.244 µg/ml and 63.7% in 0.488 µg/ml. Thus, this compound was further evaluated in differentiated SH-SY5Y cells.

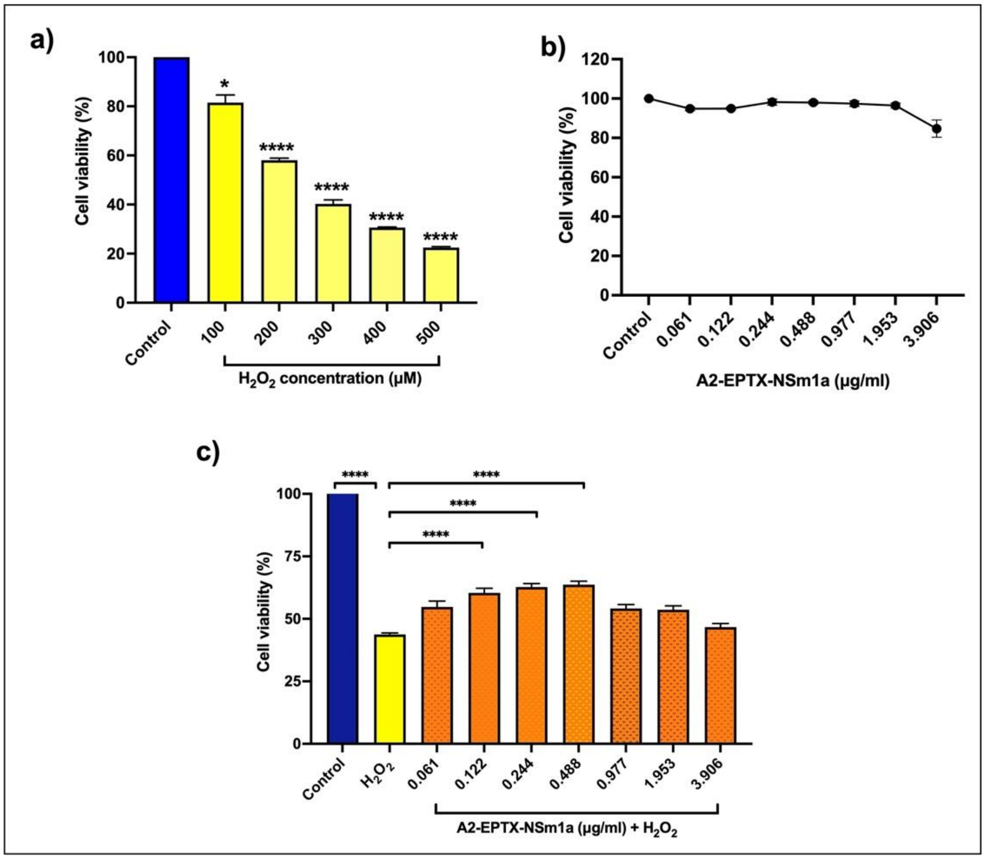

Supplementary Figure 1. (a) Cell viability of hydrogen peroxide (H_2O_2) incubated for 24 hours with concentration 100 to 500 µM in the undifferentiated neuroblastoma cell line, SH-SY5Y. (b) Cell viability of A2-EPTx-NSm1a at concentration 0 to 3.906 µg/ml for 24 hrs of incubation. (c) Neuroprotectivity activity of A2-EPTx-NSm1a in H_2O_2 induced toxicity model using neuroblastoma cell line, SH-SY5Y. Data was reported as mean ± SEM, where $p < 0.05$ is considered as significant. * indicated $p<0.05$, **** indicated $p<0.001$.

Reference:

Abdullah, N.A.H., Rusmili, M.R.A., Zainal Abidin, S.A., Shaikh, M.F., Hodgson, W.C., and Othman, I. (2021). Isolation and Characterization of A2-EPTX-Nsm1a, a Secretory Phospholipase A_2 from Malaysian Spitting Cobra (*Naja sumatrana*) Venom. *Toxins (Basel)* 13(12). doi: 10.3390/toxins13120859.
